# Adaptive optical phase estimation for real-time sensing of fast-varying signals

**DOI:** 10.1038/s41598-022-26329-1

**Published:** 2022-12-16

**Authors:** Liu Wang, Fang Xie, Yong Zhang, Min Xiao, Fang Liu

**Affiliations:** 1 Department of Physics, Nanjing Tech University, Nanjing, 211816 China; 2 National Laboratory of Solid State Microstructures, College of Engineering and Applied Sciences, and School of Physics, Nanjing University, Nanjing, 210093 China; 3 Department of Physics, University of Arkansas, Fayetteville, AR 72701 USA

**Keywords:** Optics and photonics, Physics

## Abstract

Optical phase tracking is an important technique for use in high-precision measurement applications, including optical frequency metrology and ground- or space-based gravitational wave observation, and coherent optical communications. When measuring fast-varying real-time signals, the response time limitations of the measurement system’s phase-locked loop cause the best operating point to be mismatched, and the measurement then becomes nonlinear. To make these measurements possible, this work proposes a time delay loop that theoretically enables optimal homodyne detection. When the time delay loop is combined with an extended Kalman filter, the estimated measurement accuracy is improved by 2.4 dB when tracking a fast-varying random signal with a velocity of 10^7^ rad/s. This phase estimation improvement also increases as the interference angle deviates further from the optimal measurement point. The proposed method shows potential for use in real-time sensing and measurement applications.

## Introduction

Optical phase tracking occupies a unique application position because of its use in the measurement of dynamic targets or signals^1–6^, including gravitational wave detection and biological measurements^7,8^. However, in classical optical measurement, each measurement has an upper precision limit, which is the quantum noise limit determined by quantum mechanics^9–16^. For constant phase measurements, the optical measurement accuracy limit is determined based on the number of photons *N* to be $1/\sqrt N$^10^. The main method that is used at present to exceed the optical measurement accuracy limit involves the use of nonclassical light sources^11,17–20^. For example, in 1981, Caves first proposed that the Mach–Zehnder interferometer should use squeezed vacuum light to achieve sub-shot-noise sensitivity levels^10^. For dynamic targets, Wiseman et al. proposed a feedback control measurement scheme, in which the measurement information was used to enable feedback control of the local oscillator phase; the relative phase between the local oscillator light and the signal to be measured was then locked at $\pi /2$, and it was verified that the measurement accuracy of this adaptive method is $\sqrt 2$ times that of the non-adaptive method^21^. On the basis of Wiseman’s proposed adaptive feedback measurement structure, large numbers of classical estimation theories have been used to determine the phase parameters of both coherent light and squeezed light. Among these efforts, Tsang et al. designed a zero-beat phase-locked loop that used a Kalman–Bucy filter and a Wiener filter to realize measurements of the real-time phase and the instantaneous frequency of coherent light, respectively^22^. In 2010, Wheatley et al. proposed a data smoothing scheme to track the phase of squeezed light. Experiments showed that the phase accuracy obtained was two times higher than the limit that can be reached by coherent light^23^.

In optical measurements, a great deal of this research has been used in practical applications. Xiao et al. successfully exceeded the shot noise limit accuracy using Mach–Zehnder interference in 1987^24^. In 2002, Armen et al. used an optical phase-locked loop to track optical phase continuously^25^. In 2012, optical phase tracking was also realized using squeezed light, and this approach was then used to track the movements of mirrors^26,27^. In 2019, to improve the convenience of this system further, Zhang et al. realized continuous tracking of signals in optical fibers^28,29^. Optical phase tracking of real-time signals has always been an important development direction for optical measurement and has proved to be an important technique in practice.

In previous works, the optical phase of the signal was always recorded at the optimal measurement point under the lock of a phase-locked loop^26,30–32^. In this paper, a system structure with a time delay is proposed that can solve the problem that occurs when the signal rate is too fast and the phase-locked loop is not locked at the optimal measurement point. The proposed structure makes it possible to track the optimal point for measurement of the phase difference between the signal phase and the local oscillator phase throughout the entire estimation process. In this work, we provide a theoretical explanation of the advantages of the proposed new time-delay system structure for use in fast time-varying signal phase processing. Because the first measurement is not optimal, the proposed system realizes the optimal measurement signal at the expense of some photon resources. Furthermore, we build an extended Kalman filter model for the new structure that improves both the system stability and the accuracy of the final results^33–36^. Theoretical and simulation-based analyses show that this new system structure can perform measurement and tracking of fast objects in practical applications.

## Time delay detection

**Figure 1:**
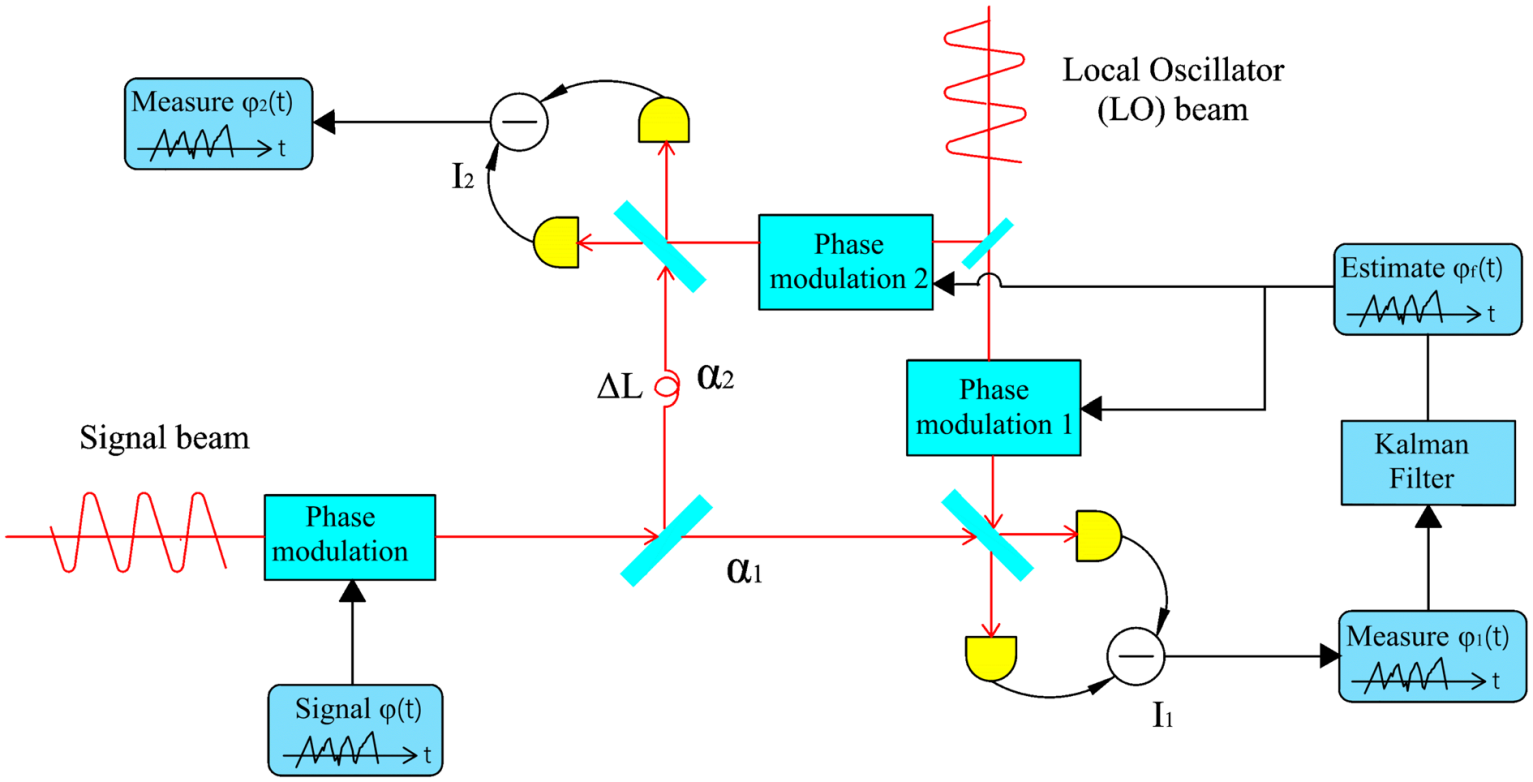
Theoretical scheme for optical phase tracking system with an optical delay loop. As illustrated in the figure, the signal phase ${\varphi }_{1}$ is measured first, and the local oscillator light is then modulated. ${\alpha }_{1}$ and ${\alpha }_{2}$ represent the amplitude operators of the signal after beam splitting. The local oscillator’s modulation and feedback time is compensated by addition of an extra optical path $\Delta L$ to achieve the time delay effect, which means that the information phase ${\varphi }_{2}$ is measured at the optimal measurement point.

At present, direct detection of the phase information carried in the optical frequency band is impossible. The most commonly used method to extract phase information is via a method that involves optical interference of two laser beams with the same operating frequency. Here, we consider the use of continuous wave interferometry, where the acquisition processes for phases $\varphi_{1}$ and $\varphi_{2}$ are as shown in Fig. 1. In this approach, a time delay measurement component is added to the traditional optical phase-locked loop. When a real-time phase $\varphi$ is carried into the signal beam, the output current from balanced detector 1 is then given by the following equation^26^:

1 $$I_{1} (t) = 2\left| {\alpha_{1} } \right|\sin \left[ {\varphi (t) - \varphi_{1} (t)} \right] + W_{1} (t),$$

where $\alpha_{1}$ is the amplitude operator of the signal beam that passes through the first optical splitter toward detector 1, and $W_{1} (t)$ is modeled as independent Gaussian white noise that satisfies the relationship $\left\langle {W_{1} (t)W_{1} (\tau )} \right\rangle = \delta (t - \tau )$. The total number of photons in this case is $\left| \alpha \right|^{2} = \left| {\alpha_{1} } \right|^{2} + \left| {\alpha_{2} } \right|^{2}$, and the ratio of the number of photons at detector 2 to the total photon number is defined by $\kappa ={\left|{\alpha }_{2}\right|}^{2}/{\left|\alpha \right|}^{2}$. Generally, to achieve the maximum sensitivity in a traditional homodyne detection system, similar to that obtained in previous works^21,26,28,29,37^, the modulation phase of the local beam is locked at $\Phi (t) = \varphi_{1} (t) + \pi /2$, where $\varphi_{1} (t)$ is obtained from the signal $\varphi (t)$, and the output current can be linearized as ${I}_{1}(t)=2\left|{\alpha }_{1}\right|\left[\varphi (t)-{\varphi }_{1}(t)\right]+{W}_{1}(t$). In this paper, we consider the case where the signal rate is changing too rapidly. When the rate of the signal $\dot{\varphi }$ changes too quickly, the feedback time $\delta$ of the PLL cannot be ignored, and the condition that $\langle {\left[\varphi (t)-{\varphi }_{1}(t-\delta )\right]}^{2}\rangle \ll 1$ cannot be met. For example, if the signal beam phase varies as $\dot{\varphi }\tau = \pi /4$, then the sensing coefficient will decrease by 30%, and the measurement error will increase by 40% because of the time delay of the PLL. Therefore, when the signal rate changes too rapidly and the PLL feedback time $\delta$ is taken into account, the output current from balanced detector 1 should be:

2 $${I}_{1}\left(t\right)=2\left|{\alpha }_{1}\right|{\sin}\left[\varphi \left(t\right)-{\varphi }_{1}\left(t-\delta \right)\right]+{W}_{1}\left(t\right).$$

The signal beam input to detector 2 is loaded with an extra phase by delaying it for a distance denoted by $\Delta L$. If the required time meets the feedback time $\delta$ exactly, then the local phase is synchronized with the signal phase. Different from the nonlinear measurement of detector 1, detector 2 gives a linear output because it is always at the optimal measurement point, i.e., it can be considered that the relationship ${\sin}(\varphi -{\varphi }_{1})\approx \varphi -{\varphi }_{1}$ approximately holds. The output current of balanced detector 2 then defaults to

3 $${I}_{2}\left(t\right)=2\left|{\alpha }_{2}\right|\left[\varphi \left(t\right)-{\varphi }_{1}\left(t\right)\right]+{W}_{2}\left(t\right).$$

$W_{2} (t)$ is modeled as independent Gaussian white noise here and satisfies the relationship $\left\langle {W_{2} (t)W_{2} (\tau )} \right\rangle = \delta (t - \tau )$. In this case, the measurement signals for detectors 1 and 2 are named $\varphi_{1} (t)$ and $\varphi_{2} (t)$, respectively. Note that sufficient photon resources must be provided for the first measurement to ensure that the second measurement will not deviate from the best working point. In this paper, it is assumed that the deviation angle $\Delta \varphi$ of the error of detector 1 does not exceed 0.017 rad (which is approximately a 1° deviation from the optimal working point) in the second measurement. For a given phase error $\varphi ={\varphi }_{1}(t)-\varphi (t)$ for detector 1 and a sensing coefficient of $2\left|{\alpha }_{1}\right|\mathrm{cos}\left[\varphi (t)-{\varphi }_{1}(t-\delta )\right]$, according to the “3*σ*” principle of a normal distribution, the number of photons received by detector 1 should satisfy the relationship ${\left|{\alpha }_{1}\right|}^{2}\ge {\left\{0.0113\cdot \mathrm{cos}\left[\varphi (t)-{\varphi }_{1}(t-\delta )\right]\right\}}^{-2}$.

In this paper, the results of detector 1 and detector 2 can’t be ignored. Our final result ${\varphi }_{s}$ is obtained from the results of detector 1 and detector 2, that is, $\varphi_{s} = \left[ {I_{1} \left| {\alpha_{1} } \right|\cos (\varphi - \varphi_{1} } \right) + I_{2} \left| {\alpha_{2} } \right|]\left[ {2\left| {\alpha_{1} } \right|^{2} \cos^{2} (\varphi - \varphi_{1} ) + 2\left| {\alpha_{2} } \right|^{2} } \right]^{ - 1}$^38^. At the same time, the mean square error (MSE) of the time delay detection results which varies with the offset angle and the optical splitting ratio is obtained by combining twice measurement result using probability theory, so that the MSE satisfies the relationship $\sigma^{2} { = }\left\{ {4\left| \alpha \right|^{2} \left[ {\kappa + (1 - \kappa )\cos^{2} (\varphi - \varphi_{1} )} \right]} \right\}^{ - 1}$^38^.

**Figure 2:**
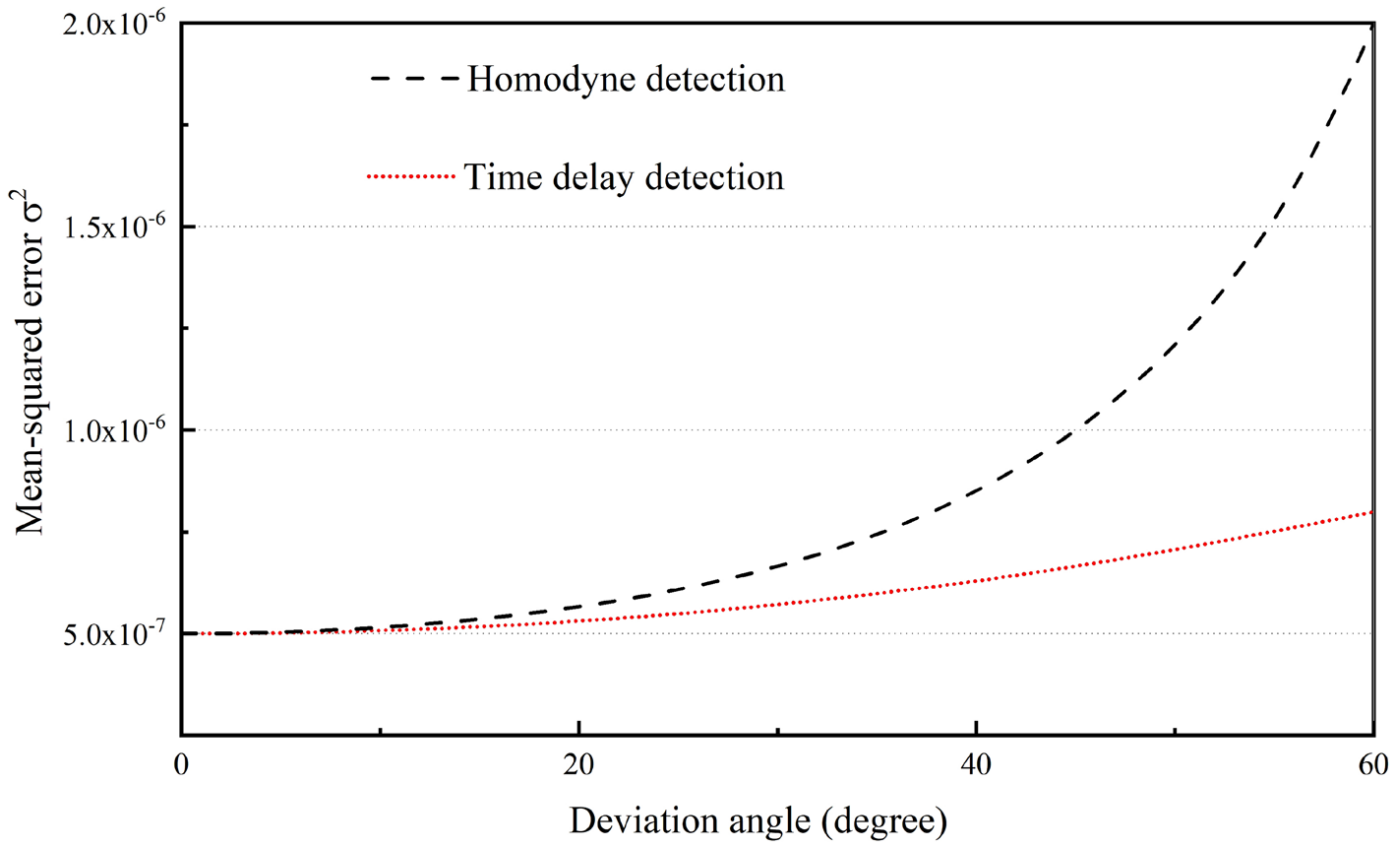
Comparison of the measurement accuracies of homodyne detection and time delay detection when the interference angle deviates from the optimal measurement point and the total photon flux remains the same at $\left| \alpha \right|^{2} = 0.5 \times 10^{6}$.

**Figure 3:**
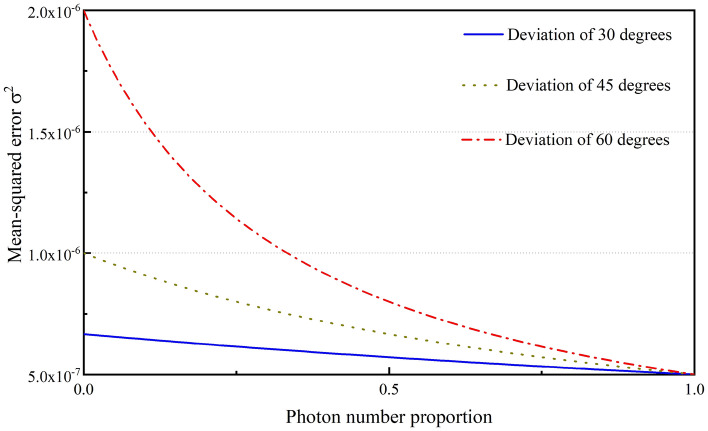
Dependence of MSE *σ*^2^ on the ratio of the number of photons at detector 2 in the time delay detection, where the ratio of the number of photons is $\kappa ={\left|{\alpha }_{2}\right|}^{2}/{\left|\alpha \right|}^{2}$.

Because the optical splitting ratio is set at 50/50, it is also important to measure the photon resources that are used for the first measurement. In Fig. 2, the mean square error (MSE) of the time delay detection result is obtained by combining the first measurement result with the second measurement result using probability theory in such a manner that the MSE satisfies the relationship ${\sigma }^{2}={\left\{2{\left|\alpha \right|}^{2}\left[1+{\mathrm{cos}}^{2}(\varphi -{\varphi }_{1})\right]\right\}}^{-1}$^38^. We also studied the estimated sensitivity corresponding to the optical splitting ratio for the signal beam between the first and second measurements. As shown in Fig. 3, when the first measurement deviates by 30°, 45°, and 60°, the overall accuracy of the entire measurement system improves as the division ratio for the first measurement decreases.

## Kalman filter

We track a random moving signal here, and $\Delta t$ is the measurement interval of the detector, which is determined by the photodetector’s bandwidth. When the input signal simulates the random fluctuations of objects, we obtain^39^:

4 $$\dot{\varphi }(k) = \dot{\varphi }(k - 1) + w(k - 1)\Delta t,$$

5 $$\varphi (k) = \varphi (k - 1) + \dot{\varphi }(k - 1)\Delta t + w(k - 1)\frac{{\Delta t^{2} }}{2},$$

where $\varphi$ is a variable that describes the angular of an object, $\dot{\varphi }$ is a variable that describes the angular velocity of that object, and $w$ represents a random disturbance of the external environment, which is an independent Gaussian white noise environment that satisfies the relationship $E\left[ {w(k)w^{T} (j)} \right] = Q\delta_{kj}$.

The equation of motion state of an object with $X(t) = [\varphi (t),\dot{\varphi }(t)]^{T}$ can be abbreviated in the form

6 $$X(k) = AX(k - 1) + Bw(k - 1),$$

where $$A = \left[ {\begin{array}{*{20}c} 1 & {\Delta t} \\ 0 & 1 \\ \end{array} } \right]$$ and $$B = \left[ {\begin{array}{*{20}c} {\frac{{\Delta t^{2} }}{2}} \\ {\Delta t} \\ \end{array} } \right]$$.

In real applications, because of the slow modulation speed of modulator 1, the bandwidth of the photodetector is much greater than that of modulator 1, and the observation equation for balanced detector 1 is thus given as:

7 $$I_{1} (k) = 2\left| {\alpha_{1} } \right|\sin \left[ {\varphi (k) - \varphi_{1} (k^{^{\prime}} )} \right] + W_{1} (k).$$

$W_{1} (k)$, which is the Gaussian white noise, is caused by shot noise, and satisfies the relationship $E\left[{W}_{1}(k){W}_{1}^{\rm T}(j)\right]={\delta }_{kj}$, ${k}^{^{\prime}}=M-N$, and *N* is a multiple of the detector bandwidth relative to the bandwidth of modulator 1, i.e., it is the number of data points acquired by that detector within the modulation interval of modulator 1. The feedback point for each modulation interval is then given by $M = \left[ \frac{k}{N} \right] \times N$.

In a continuous discrete Kalman system, if the optimal evaluation is $\overline{X} (k)$ and its error covariance matrix is $\Sigma (k) = E\left[ {(X(k) - \overline{X} (k))(X(k) - \overline{X} (k))^{T} }\right]$, then:

8 $$\overline{{X^{^{\prime}} }} (k) = A\overline{X} (k - 1),$$

9 $$\overline{\Sigma } (k) = A\Sigma (k - 1)A^{T} + Q.$$

Next, the following equation must be solved:

10 $$I_{1} (k) - 2\left| {\alpha_{1} } \right|\sin \left[ {\varphi_{1} (k) - \varphi_{1} (k^{^{\prime}} )} \right] = 0.$$

Here, we obtain $\varphi_{1} (k)$, let $H(k) = 2\left| {\alpha_{1} } \right|\cos \left[ {\varphi_{1} (k) - \varphi_{1} (k^{^{\prime}} )} \right]$, and then execute the update step using the following rules:

11 $$\overline{X} (k) = A\overline{X} (k - 1) + K(k)\overline{y} (k),$$

12 $$\Sigma (k) = [1 - H(k)K(k)]\overline{\Sigma } (k).$$

Subsequent calculations of the innovation $\overline{y}$ and the Kalman gain $K$ are then dependent on the new observation $I_{1}$, where

13 $$\overline{y} (k) = I_{1} (k) - \overline{I} (k),$$

14 $$K(k) = H(k)\overline{\Sigma } (k)S^{ - 1} (k).$$

Here, $\overline{I} (k) = H(k)\overline{{X^{^{\prime}} }} (k)$ represents the Kalman estimation value observed at a time $k$, and its accuracy is quantified using the following covariance matrix:

15 $$S(k) = E\left[ {\overline{y} (k)\overline{y}^{T} (k)} \right] = R_{1} + H(k)\overline{\Sigma } (k)H^{T} (k).$$

Here, $R_{1} = 1$. Because the system is nonlinear, an extended Kalman filter is used here. The previous part of the theory represents only the optimization of the feedback part of the system. Because of the fast causal estimation property of the Kalman filter, we also applied an extended Kalman filter to the final result acquired from the time delay system. The phases measured by detector 1 and detector 2 are $\varphi_{1}$ and $\varphi_{2}$, respectively, and the final signal can be then obtained based on the following combination of their mathematical probabilities^38^:

16 $$\varphi_{s} (k) = \left\{ {\varphi_{1} (k)\left| {\alpha_{1} } \right|^{2} \cos^{2} \left[ {\varphi_{1} (k) - \varphi_{1} (k^{^{\prime}} )} \right] + \varphi_{2} (k)\left| {\alpha_{2} } \right|^{2} } \right\}/\left\{ {\left| {\alpha_{1} } \right|^{2} \cos^{2} \left[ {\varphi_{1} (k) - \varphi_{1} (k^{^{\prime}} )} \right]) + \left| {\alpha_{2} } \right|^{2} } \right\}.$$

Here, $\varphi_{s} (k)$ represents the result of the comprehensive measurements of detector 1 and detector 2. Because modulator 2 can modulate the signal with a high bandwidth, the observation coefficient is always $\left| {2\alpha_{2} } \right|$. We apply a Kalman filter again here. The observation coefficient is set at $\left| {2\alpha } \right|$, the observation noise is set at $2R_{1} /\left\{ {\cos^{2} \left[ {\varphi_{1} (k) - \varphi_{1} (k^{^{\prime}} )} \right] + 1} \right\}$, and the Kalman filter value of the synthesis $\varphi_{s} (k)$ can then be obtained using the method described above.

## Simulation of time-varying phase tracking

**Figure 4:**
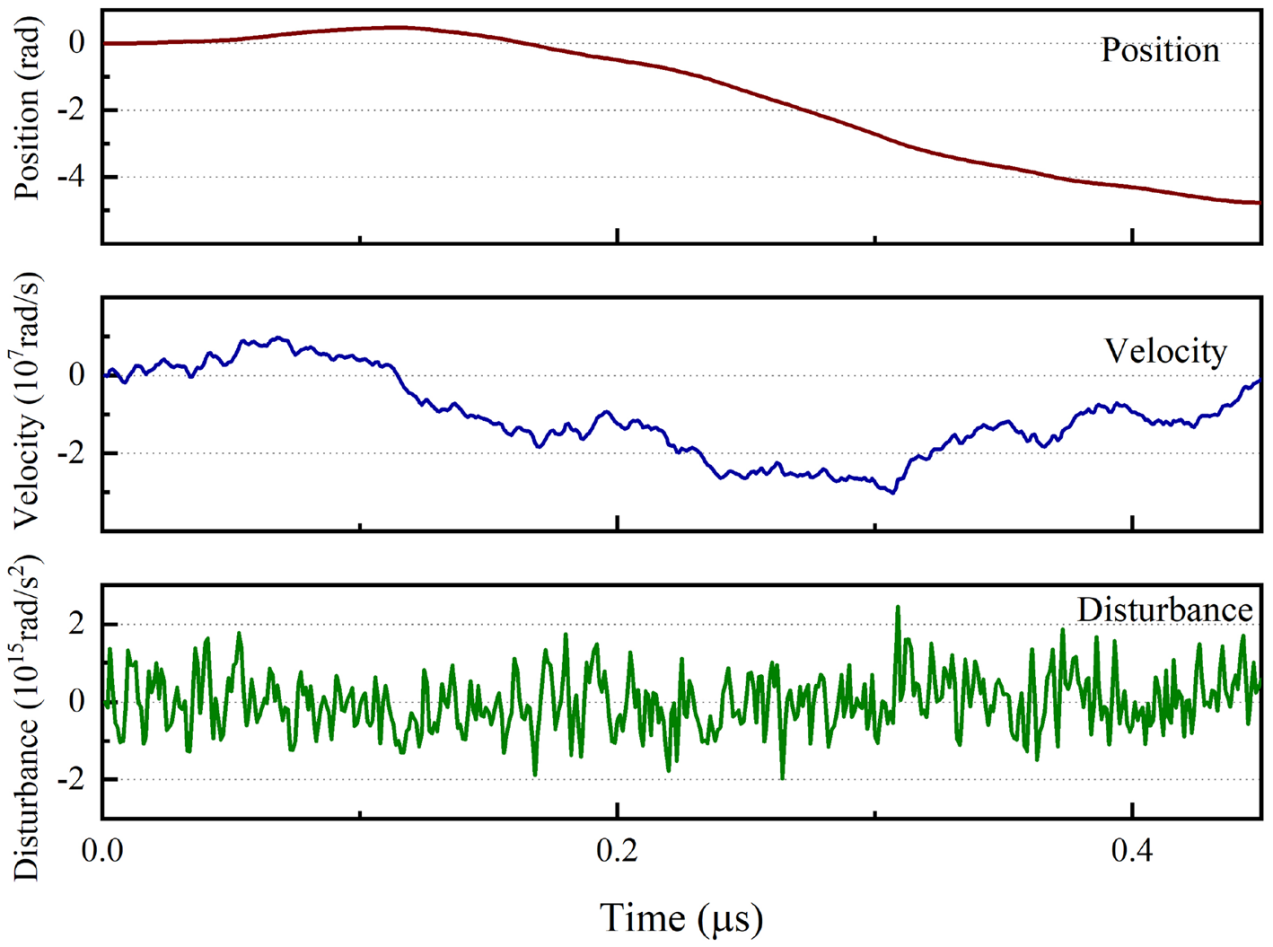
Time traces of the object motion signals. The position, velocity, and acceleration (disturbance) characteristics of the motions caused by random perturbations are shown by these traces.

In this paper, discrete signals are used to perform the simulations, and the bandwidths of the photodetectors and the phase-locked loops are both set. It is assumed here that the photodetector bandwidth is 1 GHz, the bandwidth of modulator 1 is 40 MHz, the bandwidth of modulator 2 is 1 GHz, and the feedback delay is 25 ns^40^. Figure 4 shows a generated random displacement graph. In this paper, the working phase speed is at a level of approximately 10^7^ rad/s, as compared with the level of only 10^5^ rad/s used in a previous work^27^. In the figure, “disturbance” represents the acceleration of an object caused by a random external force, and the magnitude of the disturbance is determined by the signal noise $Q$. Next, we track the phase information using both the new time-delay structure (with the 50/50 light splitting ratio) proposed in Fig. 1 and the traditional classical phase-locked loop. The optimal measurement point for the signal offset is located within the range from 0–1.22 rad.

**Figure 5:**
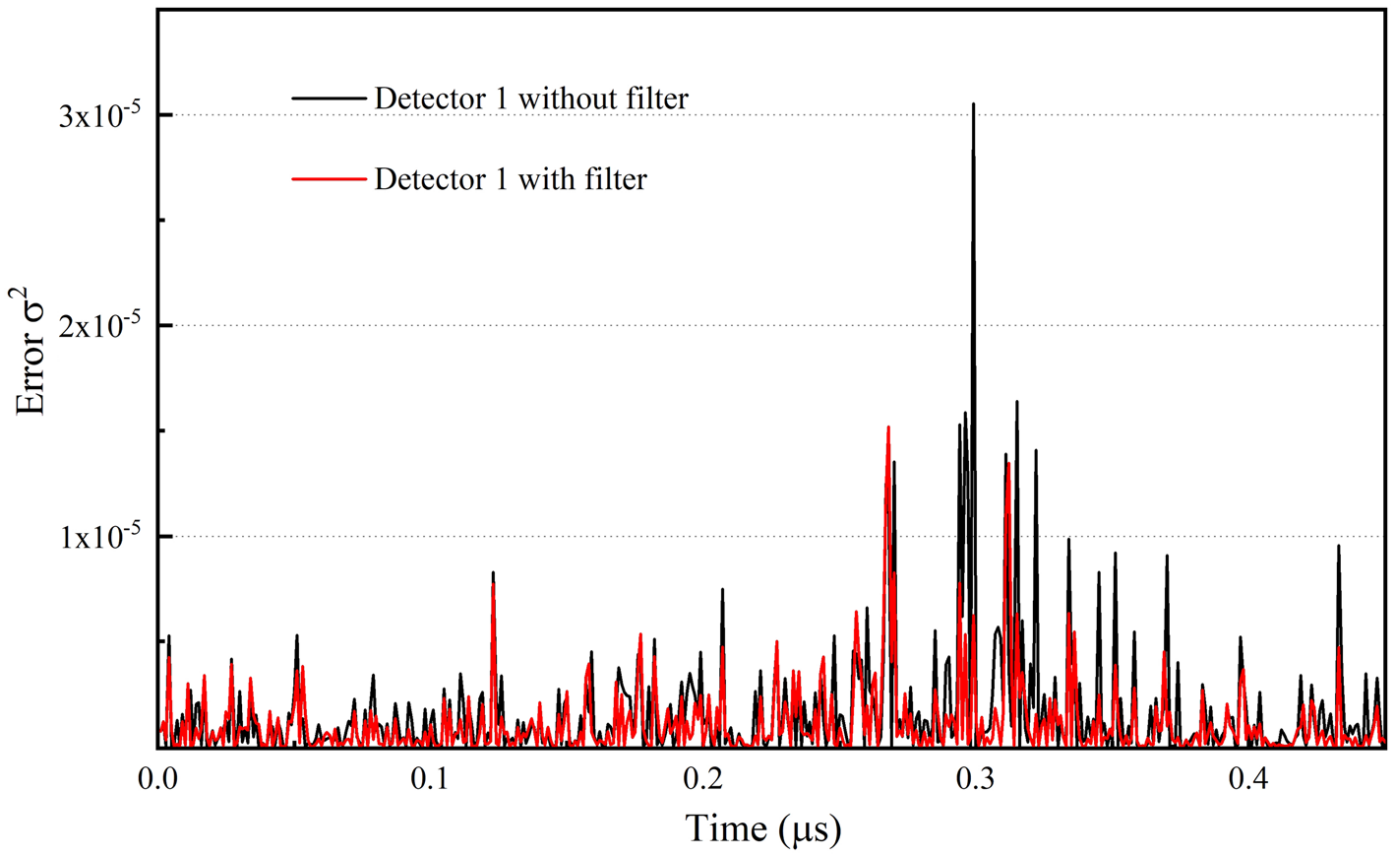
Phase variation characteristics of a random signal with and without Kalman filtering.

To demonstrate the improvement in the phase tracking obtained with the consideration of the Kalman filter, the phase variation in the random signal illustrated in Fig. 4 is measured by homodyne detection at detector 1. It is observed that the fluctuations in the measured parameter are reduced with the aid of the extended Kalman filter, as illustrated in Fig. 5, where the total photon flux $\left| \alpha \right|^{2} = 0.5 \times 10^{6}$, the signal noise $Q = 10^{ - 6}$, and the error $\sigma^{2} = (x - \varphi )^{2}$, where $\varphi$ is the input phase, and $x$ is the measured or filtered value. The maximum angle deviation caused by the first measurement error in this simulation is 0.014 rad, and the fluctuation of observation coefficient for this deviation is less than $1{0}^{-4}$. Here, the feedback component of the PLL is estimated using the extended Kalman filter based on the discussion above. Additionally, it is necessary to assess the effect of the Kalman filter with respect to the MSE. Based on consideration of the data, which were sampled 10^5^ times, the MSE given by the direct measurement without the Kalman filter is $1.57 \times 10^{ - 6}$, and the corresponding MSE with the Kalman filter is $1.05 \times 10^{ - 6}$. Therefore, when the extended Kalman filter is implemented for real-time phase estimation, the estimation accuracy is optimized by 1.7 dB.

**Figure 6:**
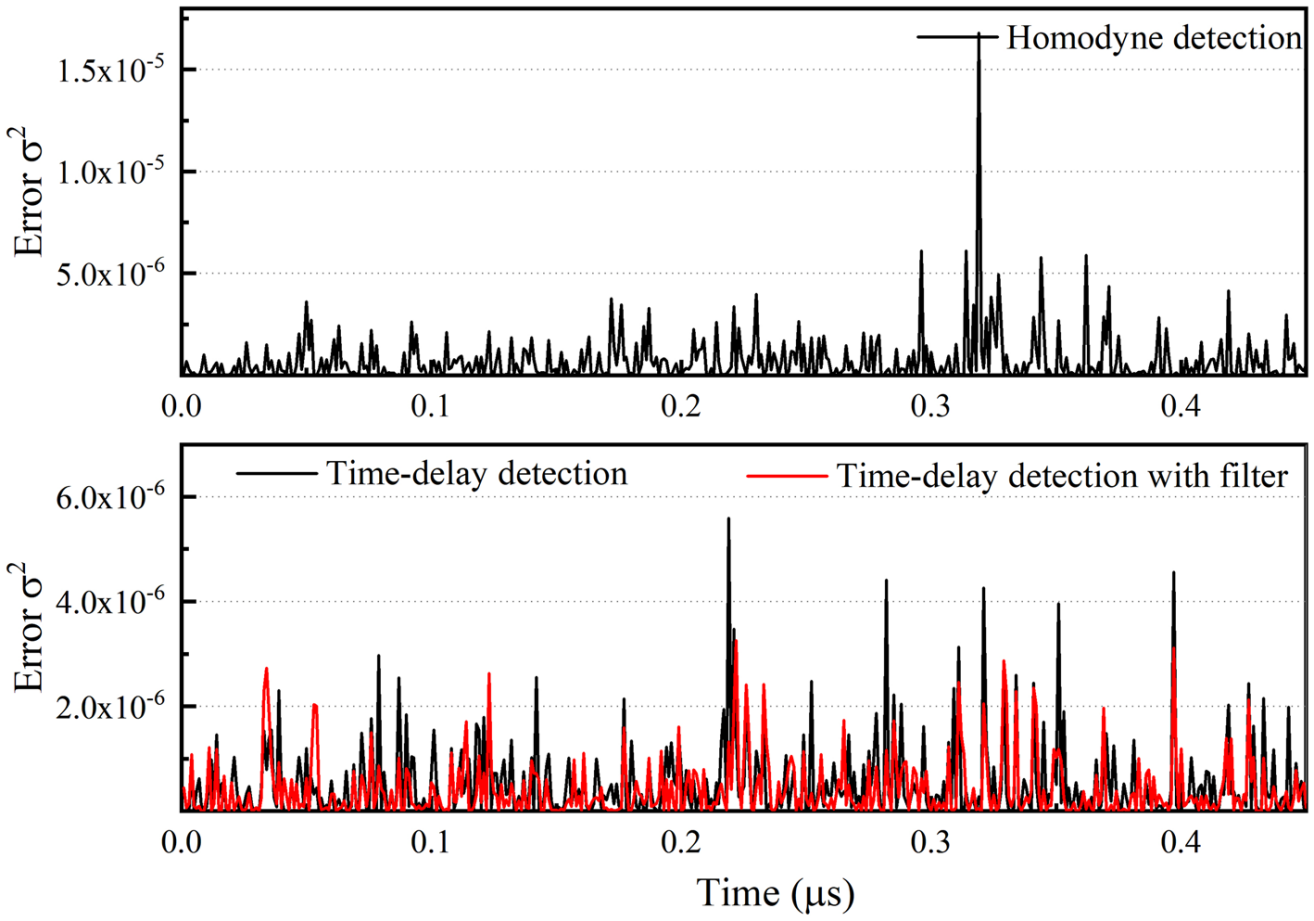
Comparison chart for results of classical direct structure measurement, delay structure measurement, and delay structure measurement with additional filtering.

Finally, the phase sensitivity performance produced by application of the new time-delay structure is discussed. As shown in Fig. 6, when compared with the results from conventional homodyne detection, the measured phase variation is obviously reduced when the time-delay structure is used. The enhancement effect can also be characterized using the MSE. The MSE of the time-delay measurement in this case is $5.29 \times 10^{ - 7}$, which is close to the theoretical limit of $5 \times 10^{ - 7}$ determined under the conditions at the optimal operating point for a total photon flux $\left| \alpha \right|^{2} = 0.5 \times 10^{6}$, while an MSE of $7.03 \times 10^{ - 7}$ was measured when using conventional homodyne detection. Furthermore, the introduction of the Kalman filter to the phase estimation process causes the MSE to decrease to $4.06 \times 10^{ - 7}$, and the phase accuracy is therefore enhanced by a factor of 2.4 dB. Additionally, although this enhancement continues to increase as the interference angle deviates more widely from the optimal measurement point, as shown in Fig. 2, we must consider the average effect here and obtain the overall optimization when tracking a random signal.

## Conclusion

In summary, we have designed a new type of optical phase tracking system with a time delay loop that can achieve high-precision measurement of the high-speed phase variations in object motion in practical applications. When compared with conventional homodyne detection, the phase variation is reduced obviously when the system is implemented to perform time delay detection while tracking a real-time signal at high velocity; in particular, the phase enhancement improved as the deviation from the optimal operating point increased. Addition of the extended Kalman filter algorithm led to an enhancement of the measurement accuracy by a factor of 2.4 dB being obtained based on double measurements. With the ongoing developments in science and technology, our method has demonstrated its potential for application to time-varying signal sensing and dynamic measurements in the future.

## Data Availability

The datasets generated during and/or analyzed during the current study are available from the corresponding author on reasonable request.
